# Cutaneous Leishmaniasis Prevalence and Clinical Overview: A Single Center Study from Saudi Arabia, Eastern Region, Al-Ahsa

**DOI:** 10.3390/tropicalmed8120507

**Published:** 2023-11-24

**Authors:** Mahdi Al-Dhafiri, Abdulmohsen Alhajri, Zahraa Ali Alwayel, Jasmine Ahmed Alturaiki, Shaima Ali Bu Izran, Fatimah Ahmed Alhammad, Ryhana Mohammed Aljumaiah

**Affiliations:** 1 Department of Dermatology, King Faisal University, Al-Ahsa 31982, Saudi Arabia; 2 Department of Dermatology, King Fahad Hospital, Al-Ahsa 31982, Saudi Arabia; ahajri@gmail.com; 3 General Practitioner, The Ministry of Health, Al-Ahsa 31982, Saudi Arabia; 4 Medical intern, King Faisal University, Al-Ahsa 31982, Saudi Arabia; j.a.alturaiki@hotmail.com; 5 Internal Medicine Resident, King Fahad Hospital, Al-Ahsa 31982, Saudi Arabiaraihanahmtj@hotmail.com (R.M.A.); 6 Family Medicine Resident, Family Medicine Academy, Al-Ahsa 31982, Saudi Arabia; fatimah.alhammad.1@hotmail.com

**Keywords:** cutaneous leishmaniasis, endemic, Al-Ahsa, Kingdom of Saudi Arabia, PCR

## Abstract

Cutaneous leishmaniasis (CL) is a vector-borne parasitic disease that is transmitted via the bites of infected female sandflies. CL has been endemic in several countries worldwide for many decades, and numerous cases have been reported in Saudi Arabia, particularly across six regions; one of which is AL-Ahsa. Our research aimed to evaluate the epidemiological situation of CL among the patients in Al-Ahsa, Eastern region, Kingdom of Saudi Arabia, during the period from 2017 to 2023. The data were collected from the patients’ registries and included 245 patients who were diagnosed with CL in Al-Ahsa, Saudi Arabia. Fewer than half of the cases (47.8%) were Saudi patients, with a significant number of them being males (84.5%). Over half of the cases (52.7%) were aged between 21 and 40 years, and about three-quarters (74.7%) of the cases resided in rural areas. Regarding the nature of the lesions, 38.4% of the cases had one lesion, which was mainly distributed on a lower extremity (62.0%) or an upper extremity (52.2%). A high percentage of the lesions (75.2%) were wet lesions and caused by *L. major*. Concerning risk factors, a greater number of patients (71.4%) had been in rural areas within the last three months. Additionally, more than half of the cases (54.3%) had close contact with rodents, followed by birds (28.2%), sheep (22.0%), dogs (16.3%), and other animals (1.2%). The results showed a low number of reported CL cases in 2020 and 2021, followed by a surge in 2022 and 2023. The study shows that cutaneous leishmaniasis is still a public health problem in Al-Ahsa and is primarily associated with rural areas.

## 1. Introduction

Leishmaniasis is a complex of tropical neglected vector-borne diseases caused by heterogeneous species of obligate intracellular flagellated protozoan parasites belonging to the genus Leishmania, which is further divided into the subgenus *Leishmania* (L.) and *Viannia* (V.) species [1]. The vast majority of leishmaniasis cases are transmitted to humans through the bite of more than 90 species of infected female sandflies. *Phlebotomus sandflies* are responsible for Old World Leishmaniasis (OWL), which is found in Asia, the Middle East, and North Africa. *Lutzomyia sandflies* are responsible for New World Leishmaniasis (NWL), which is found in Mexico, Central America, and South America [1,2,3,4]. In accordance with the aforementioned classification, there are more than twenty identified *Leishmania* species worldwide. OWL species primarily include *L. (L.) donovani, L. (L.) infantum, L. (L.) major, L. (L.) tropica,* and *L. (L.) aethiopica*, while the NWL species are *L. (L.) amazonensis, L. (L.) mexicana L, L. (L.) chagasi, L. (V.) naiffi, L. (V.) braziliensis, and L. (V.) guyanensis*. The OWL species usually cause a self-limiting disease with a spontaneous resolution in months to years, unlike the NWL species, which tend to be severely destructive and disfiguring [5].

The estimated global incidence of leishmaniasis is 700,000 to 1.2 million cases per year [2,3]. In that regard, the World Health Organization (WHO) has recently designated leishmaniasis as among the most overlooked neglected infectious diseases on a global scale that merits exercising caution in terms of disease trend monitoring and establishing stellar surveillance systems in countries with a documented history of leishmaniasis endemicity [2]. Three main clinical forms of leishmaniasis exist, and they differ in clinical presentation. These are cutaneous leishmaniasis (CL), visceral leishmaniasis (VL), and mucocutaneous leishmaniasis (MCL) [2,6]. Our research mainly focuses on CL, which is the most prevalent type worldwide with an estimated annual incidence of 600,000 to 1 million new cases [2]. CL is a skin disorder characterized by isolated or multiple lesions of verrucous or ulcerated plaques on exposed body parts accessible to the bites of infected female sandflies. Sometimes, they are accompanied by satellite lesions and/or nodular lymphangitis [7,8].

As of 2020, while the Kingdom of Saudi Arabia (KSA) was not among the ten countries with the highest number of reported cases globally, it was still the fourth most endemic area for CL in Western Asia [9]. The emergence of CL in KSA is influenced by various factors, including high population mobility related to immigrants or visitors to the holy places of Makkah and Madinah, climate change, and environmental changes due to the rapid urbanization of reservoir habitats by humans or agricultural advancement [10].

The primary *Leishmania* species responsible for CL in KSA are *L. major* (zoonotic cutaneous leishmaniasis—ZCL), and *L. tropica* (anthroponotic cutaneous leishmaniasis—ACL). *L. major* stands to be the predominant etiological species, especially in the central (Al-Qaseem, and Riyadh) and eastern (Al-Ahsa) regions of the country. On the other hand, *L. tropica* is relatively less common and is primarily found in the southeastern regions and foci in Al-Madina, Al-Qaseem, Aseer, Ta’if, and Al-Baha [11,12,13,14]. *L. major* is primarily spread via the sandfly vector *Phlebotomus (P.) papatasi*, whereas *L. tropica* is transmitted by *P. sergenti*. Two types of desert rodents have been identified as the main reservoir mammals of CL: *Psammomys obesus* and *Meriones libycus* [12].

Although CL remains a major public health concern in KSA, the last few years have witnessed a dramatic decline in the number of registered cases compared with previous years [9,12,15,16]. This is hugely attributed to the success of the Leishmaniasis Control Program (LCP) established in 1978 by the Saudi Ministry of Health (MoH), which was mandated in response to the high prevalence of CL, as a total of 18,318 new cases were reported that year. Thus, LCP has played a crucial role in reducing the burden of leishmaniasis in the country, with significant decreases in both the cutaneous and visceral forms of the disease. According to the MoH, due to their enhanced efforts dedicated toward the eradication of leishmaniasis, the number of reported cases of CL dropped from thousands in the 1980s and 1990s to about 600 in 2021. Similarly, cases of visceral leishmaniasis (VL) decreased from 217 in 1991 to zero in 2019, 2020, and 2021. The success of the program can be credited to a combination of preventive measures, including vector and reservoir control, improved healthcare services, and public awareness initiatives [9,12,15,16].

Where CL is endemic, the appearance of skin lesions in correlation with the history given by the patients is a key factor for establishing a diagnosis, relying heavily on a high level of clinical suspicion. However, skin biopsy and sterile tissue culture along with molecular testing with polymerase chain reaction (PCR) are usually required to distinguish leishmaniasis from other dermatological diseases and confirm the diagnosis [7,8,11,17]. Although CL is a self-limiting disease, therapeutic interventions are usually required to accelerate the healing process and minimize the risk of transmission between vectors and reservoirs. The response to the treatment varies depending on the *Leishmania* species or type and other factors, such as the age of the patient, the number and the size of the lesion, the type of medication used, and the patient’s compliance [7,8].

The aim of this retrospective study was to evaluate the epidemiological trends and clinical characteristics of CL among patients living in Al-Ahsa, Eastern region, KSA, from the period between 2017 and 2023.

## 2. Materials and Methods

### 2.1. Study Design

The present retrospective study was conducted to investigate the epidemiological pattern of CL from January 2017 to April 2023 among Saudi and non-Saudi patients residing in the Al-Ahsa region, KSA. KSA is situated in Southwest Asia and is divided into 20 regions (or 13 administrative provincial regions); Al-Ahsa is one of the regions found in the southeast of KSA (between latitudes 17 and 26 and longitudes 48 and 55), and it is bordered by Qatar, the United Arab Emirates (UAE), and the Sultanate of Oman. It has an estimated land space of about 530,000 square kilometers with a population of over 1.2 million people. The region is divided into three main geographical sections—the highlands, the deserts, and the plains—in addition to marshes and valleys. The climate is typically hot and dry in the summer and cold and rainy in the winter [18].

### 2.2. Study Population

The study included all Saudi and non-Saudi patients who were sent to the referral center for reported or suspected CL cases in the Al-Ahsa region from January 2017 to April 2023. A dermatologist assessed and diagnosed the patients on the basis of the diagnostic criteria, which mainly rely on history and the clinical examination of the cutaneous lesions. The differential diagnoses included other skin infections, eczema, sarcoidosis, tuberculosis, and leprosy. In instances of diagnostic uncertainty, the diagnosis was confirmed by direct Giemsa-stained smear scrapings to reveal spindle-shaped Leishman bodies (amastigotes) and PCR assays to detect the *Leishmania* parasite. The data were then documented on paper, collected, and relayed directly to be recorded in the registries of the vector-borne diseases control unit of the region, from which the results of this study were obtained. The following parameters were considered: the demographic data of the patients (age, gender, nationality, and residency area), characteristics of the lesions (the number, type, and distribution of the lesions), and risk factors (living in a rural area or visiting rural areas within the past 3 months, living in a house devoid of reinforced doors or windows (reinforced houses typically refer to houses designed to ward off and minimize the entry of insects and pests), nearby breeding and resting sites for sandflies, a similar presentation within the patient’s contacts, and the presence of animals in the house). Any missing follow-ups or patients with incomplete data were excluded from the study.

### 2.3. Statistical Methods

The data were collected, organized, and analyzed by SPSS, version 28, and were then presented in tables or graphs in terms of frequencies and percentages.

### 2.4. Ethical Considerations

Ethical approval (48-EP-2022) was obtained from the Institutional Review Board of King Faisal University, Al-Ahsa region, and the Al-Ahsa Health Cluster. Informed consent was obtained from all participants.

## 3. Results

### 3.1. The Sociodemographic Characteristics of the Study Participants

An epidemiological survey was undertaken with a total of 245 registered cases of CL in Al-Ahsa region, KSA, in the span of six years, from January 2017 to April 2023. As shown in Table 1, approximately half of the included cases (47.8%) were Saudi nationals, whereas 52.2% of them were foreign expatriates. Most of these patients were males (84.5%), while only a small fraction of them were females (15.5%). Approximately half (52.7%) of the patients fell within the age range of 21 to 40 years, followed by patients who were aged between 41 and 60 (22.7%), 20 or younger (19.5%), and finally 61 or older (3.3%). Furthermore, about three-quarters (74.7%) of the patients resided in rural areas compared with 25.3% who resided in urban areas.

### 3.2. The Characteristics of CL Lesions among the Study Participants

A total of 38.4% had one lesion, 24.1% had two lesions, 13.5% had three lesions, and 24.1% had more than three lesions. The distribution of the lesions was mainly in the lower extremities (62.0%) and upper extremities (52.2%) followed by the head (6.5%) and trunk (2.4%). Moreover, the lesions were found to be predominantly wet (75.2%), whereas 20.6% were dry; 4.2% were superinfected with bacteria, resulting in transparent to pus-like white-yellowish discharge. Nearly all the cases (93.9%) were diagnosed clinically with a high index of suspicion, and they were subsequently confirmed through PCR testing. The results distinguished *L. major* to be the causative agent in patients presenting with wet lesions, while *L. tropica* was identified as the culprit in subjects with dry lesions. (Table 2).

### 3.3. The Response of CL Lesions to Proposed Treatment Options

A greater number of patients (71.4%) had been in rural areas within the past three months (Table 3). The sources of infection were traced back to rural areas and farms (76.7% and 50.6%, respectively). Additionally, 91.0% had no similar presentation among their contacts, and 51.4% lived in a reinforced house, but 14.7% lacked reinforced windows. Furthermore, more than half of the cases (54.3%) had close contact with rodents, while 28.2%, 22%, 16.3%, and 1.2% had contact with birds, sheep, dogs, and other animals, respectively. Nearby breeding sites for flies were found to originate from burrows (62%), followed by approximately 28% originating from garbage (28.6%) and 25.3% originating from corrals. Our patients were managed according to Saudi MoH guidelines for treating CL. Although CL can heal spontaneously, early intervention was prioritized for all patients to prevent disfiguring scars. Remarkably, 71.4% of infected persons achieved complete healing of the skin lesions through proper treatment. The treatment encompassed a combination of sodium stibogluconate (Pentostam^®^) injections and/or systemic antifungals, along with adjuvant therapy involving cryotherapy or the topical application of antifungals, iodine, or emollients. The rest of the patients (28.6%) either did not have regular follow-ups or failed to respond well to treatment (Table 2).

### 3.4. The Epidemiological Trend of CL in the Al-Ahsa Region

**Figure 1:**
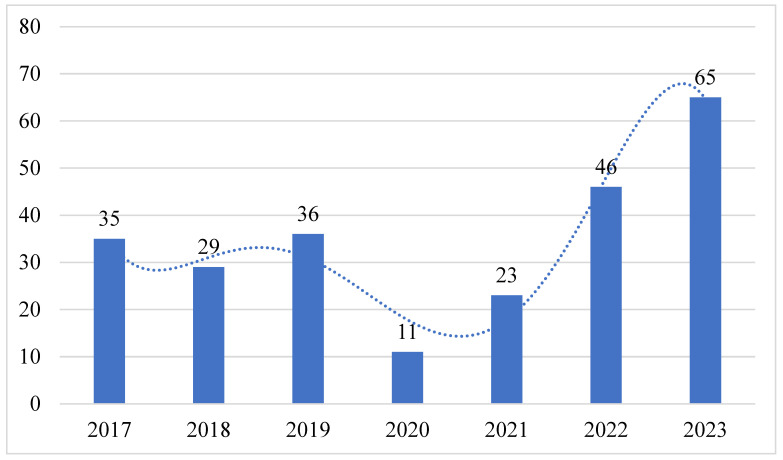
The distribution of cutaneous leishmaniasis cases by year. The *y*-axis represents the years included in the study; the *x*-axis represents the number of reported cases; the blue dot lines and columns represents how the number of cases had fluctuated over the years.

The results showed a low number of reported CL cases in 2020 and 2021, followed by a surge in 2022 and 2023. (Figure 1).

## 4. Discussion

Leishmaniasis is an important neglected tropical disease worldwide [19,20]. It imposes a significant burden on the country in terms of the financial costs associated with prevention and treatment and on infected individuals by causing remarkable psychosocial disturbances that may result from severe infections [11,19,20]. The first cases of CL in the Arabian Peninsula, in particular KSA, were officially reported in 1973, with 90 cases and an incidence rate of 100 cases per 100,000 population [15]. The highest number of reported cases come from Al-Qaseem and Riyadh (central), Al-Ahsa oasis (east), Aseer (southwest), and Ha’il and Al-Madinah (northwest), with minor variations in incidence rates over the years [10].

According to the results obtained, and among the 245 patients included in this study, a significant number of these cases (84.5%) were males. This observed higher prevalence of CL infection in males aligns with a previous study conducted in the western region of Saudi Arabia with a total of 467 patients, 406 of which were males, while a markedly smaller subset consisted of only 61 female patients [21]. Similar trends were also documented in Ethiopia, which reported an overall CL prevalence of 22.4% in the population in 2019 (males, 13.7%; females, 8.8%) [22], in addition to Yemen (males, 19.3%; females, 18.40%) [23], Libya (males, 54%; male: female ration of 1.17:1) [24], and Iraq (males, 54.6%; females, 45.4%) [25]. One plausible explanation for this gender skew in the disease distribution is that women’s practice of covering their bodies with Hijab (Islamic attire) may offer protection, whereas men’s outdoor activities during nighttime increase their exposure to sandflies [10,26]. Additionally, the incidence of CL was found to be more prevalent in foreign patients (52.2%) than in Saudi patients (47.8%). The elevated proportion of cases among non-Saudi participants could be attributed to their occupational activities and the nature of their work environment.

Our study findings revealed that approximately three-quarters (74.7%) of the patients lived in rural areas. This observation is consistent with findings from previous research conducted in Ethiopia, where 60% of the study population resided in rural areas [22]. A similar pattern was also observed in studies conducted in Yemen among patients living in villages of the Hajjah governorate [27] and in Iran, where the prevalence of CL was higher among rural residents by 56.9% [28], underscoring a higher prevalence of CL in rural regions. This correlation may be due to the risk factors prevalent in rural areas, such as the high number of domestic animals, unsanitary conditions, and poverty, all of which provide favorable habitats for sandflies [29]. Furthermore, ZCL varies according to the strain of the parasite and its hosts. The present study demonstrated that *L. major* was the causative parasite strain in most patients (71.4%) who had been in rural areas or visited farms. These findings align with the results of studies in the Al-Taif region, Saudi Arabia, which highlighted the prevalence of CL in rural areas where there is increased contact with vectors and wild animals serving as potential reservoirs [30]. On the contrary, a study in Turkey reported that the primary reservoir hosts for CL infection caused by *L. infantum* are dogs [31].

In terms of the age distribution, it is evident that more than half of the patients (52.7%) fell within the age range of 21 to 40 years. This notable trend mirrors similar observations in prior research conducted in different countries. In Ethiopia, CL caused by *L. aethiopica* was reported most commonly among individuals aged 16 to 45 years old [22]. In Sri Lanka, *L. donovani* was the primary parasite strain among patients, with a higher prevalence in the younger population aged between 21 and 40 years old [32]. Additionally, Iran reported that an extensive 70% of the patients were aged between 21 and 40 years old, although the exact type of parasite was not specified [28]. These consistent observations suggest that this age group faces a higher risk of acquiring CL infection. However, this finding contrasts with findings from studies that reported a higher prevalence of CL among younger age groups. A 46-year retrospective study that was carried out in the eastern region of Saudi Arabia in 2004 examined the epidemiological profile during the period of 1956–2002, and the findings revealed that a substantial number of cases were concentrated in the Al-Ahsa region, with a significant number of these cases occurring in patients younger than 15 years old (76%) and a peak in patients in the age bracket of one to four years old [15]. Moreover, two separate studies conducted in Yemen documented that CL had a higher prevalence rate among patients aged 1 to 15 years old and 5 to 15 years, respectively, with *L. tropica* identified as the causative agent [23,27]. Similar results were also reported in Iraq, where a significant number of cases were younger than 15 years old [25], and in Iran, where CL cases caused by *L. major* were most frequent among patients younger than 10 years old [33]. This discrepancy in CL prevalence between different age groups could be attributed to the increased exposure of younger generations to sandflies, primarily due to their active engagement in outdoor activities.

In this study, it was found that a substantial proportion of the lesions (75.2%) were of the wet type, consistent with previous research carried out in Iraq, reporting that 51.5% of patients had wet-type lesions [25]. Conversely, dry lesions were most commonly reported in the Hajjah governorate, northwest Yemen (98.6%) [27]; Anuradhapura Teaching Hospital, Sri Lanka (66%) [32]; and Borujerd county, Western Islamic Republic of Iran (57.7%) [28].

The distribution of the lesions mainly involved the lower extremities (62.0%) and upper extremities (52.2%), followed by the head (6.5%) and trunk (2.4%). These results are consistent with the findings from a previous study in Sudan, in which the lower limbs were involved in 66% of the patients and the upper limbs were involved in 50% of the cases [34], and in Iraq, in which most of the lesions were found on both the upper and lower limbs (48.8%) [25]. In contrast to previous studies conducted in Ethiopia [22] and Sri Lanka [35] that reported the head and neck to be the most commonly involved locations, in the western and southwest regions of Iran [28,31] and Shara’b District, Taiz, Yemen [23], the upper limb was the most common site of involvement for CL lesions among infected inhabitants. The finding that CL lesions are mainly distributed on the extremities can be explained by the fact that sandflies usually travel close to the ground and bite uncovered body parts, especially the lower extremities. Additionally, sleeping outdoors and constant exposure of the extremities may encourage sandflies to bite during the night [15,36].

Among the patients, 38.4% had one lesion, 24.1% had two lesions, 13.5% had three lesions, and 24.1% had more than three lesions. The prevalence of the patients with a single lesion (38.4%) aligns with the results of previous studies in Saudi Arabia (71%) [15], Iraq (49.3%) [25], Sri Lanka (77%) [30], and Iran (61.3%) [28], in which most patients presented with a solitary lesion.

The findings presented here serve to complement and expand on the earlier epidemiologic studies of CL among patients residing in the Al-Ahsa region, KSA. Our current research, however, noted a low prevalence of CL in 2020 and 2021 followed by an upsurge in 2022 and 2023. This observed variation in the incidence rate may be attributed to the impact of Coronavirus Disease 2019 (COVID-19) or the lack of reported cases owing to the circumstances. Due to the imposition of lockdown strategies, individuals were mandated to be confined in their homes during this period [37]. However, soon after the curfew was lifted, the diagnostic and screening programs that had been meticulously designed to break the leishmaniasis cycle had already been disrupted [38]. The study’s strength lies in the fact that it involved seven years of epidemiological research in an endemic area in correlation with the effect of the curfew on lowering the number of reported cases when people were not exposed to sandfly vectors or reservoir hosts. Nonetheless, it is imperative to acknowledge certain limitations inherent in this study, notably that it is retrospective and was limited to a single medical center.

## 5. Conclusions

CL persists as an endemic disease in the Al-Ahsa region, with a noteworthy upsurge in the number of registered cases in 2022 and 2023 following a period of reduced incidence during the COVID-19 lockdown period. The bulk of patients were males, non-Saudi nationals, and aged between 21 and 40. Most of them either lived or had been to rural areas in the past three months and had close contact with domestic animals. The lesions were mainly distributed on the extremities and mostly presented as single or multiple wet lesions. The program initiated by the Saudi MoH for preventing leishmaniasis might be a successful solution to reducing the number of cases, which could be explored further in future studies to gain a more comprehensive understanding of the epidemiology and risk factors of CL, thus helping to minimize the number of reported cases in endemic regions.

## Figures and Tables

**Table 1:** Sociodemographic characteristics of leishmaniasis cases (*n* = 245).

Variables		Frequency (%)
Nationality	Saudi	117 (47.8%)
	Non-Saudi	128 (52.2%)
Gender	Male	207 (84.5%)
	Female	38 (15.5%)
Age	≤20	48 (19.5%)
	21–40	129 (52.7%)
	41–60	60 (22.7%)
	≥61	8 (3.3%)
Living area	Rural area	183 (74.7%)
	Urban area	62 (25.3%)

**Table 2:** Characteristics of CL among study participants (*n* = 245).

Variables		Frequency (%)
Diagnosed by	Clinical and investigation	230 (93.9%)
	Clinical	15 (6.1%)
Number of lesions	One lesion	94 (38.4%)
	Two lesions	59 (24.1%)
	Three lesions	33 (13.5%)
	More than three lesions	59 (24.1%)
Distribution of lesions	Upper extremities	128 (52.2%)
	Lower extremities	152 (62.0%)
	Trunk	6 (2.4%)
	Head	16 (6.5%)
Types of lesions	Wet	179 (75.2%)
	Dry	49 (20.6%)
	Secondarily infected	10 (4.2%)
Therapeutic outcome	Complete healing	175 (71.4%)
	No healing	70 (28.6%)

**Table 3:** Risk factors of CL among study participants (*n* = 245).

Variables		Frequency (%)
Last place the patient had been within the past 3 months	Farms	129 (52.7%)
	Desert	47 (19.2%)
	Urban areas	55 (22.4%)
	Rural areas	175 (71.4%)
Possible origin of infection	Farms	124 (50.6%)
	Desert	38 (15.5%)
	Urban areas	58 (23.7%)
	Rural areas	188 (76.7%)
Similar presentation within the surroundings	No	232 (91.0%)
	Yes (relatives)	10 (4.1%)
	Yes (family)	8 (3.3%)
	Yes (colleagues)	4 (1.6%)
Did the patient live in a reinforced house?	Yes	126 (51.4%)
	No	146 (48.6%)
Did the house have reinforced windows?	Yes	209 (85.3%)
	No	36 (14.7%)
Nearby breeding sites for flies	Burrows	152 (62.0%)
	Garbage	70 (28.6%)
	Corrals	62 (25.3%)
Presences of animals in the house	Sheep	54 (22.0%)
	Rodents	133 (54.3%)
	Birds	69 (28.2%)
	Dogs	40 (16.3%)
	Others	3 (1.2%)

## Data Availability

All data supporting the study findings are included in this published article.
